# In vivo evolution to echinocandin resistance and increasing clonal heterogeneity in *Candida auris* during a difficult-to-control hospital outbreak, Italy, 2019 to 2022

**DOI:** 10.2807/1560-7917.ES.2023.28.14.2300161

**Published:** 2023-04-06

**Authors:** Giulia Codda, Edward Willison, Laura Magnasco, Paola Morici, Daniele Roberto Giacobbe, Antonella Mencacci, Daniele Marini, Malgorzata Mikulska, Matteo Bassetti, Anna Marchese, Vincenzo Di Pilato

**Affiliations:** 1 Department of Surgical Sciences and Integrated Diagnostics (DISC), University of Genoa, Genoa, Italy; 2 Microbiology Unit, IRCCS Ospedale Policlinico San Martino, Genoa, Italy; 3 Infectious Diseases Unit, IRCCS Ospedale Policlinico San Martino, Genoa, Italy; 4 Department of Health Sciences (DISSAL), University of Genoa, Genoa, Italy; 5 Medical Microbiology, Department of Medicine, University of Perugia, Perugia, Italy; 6 Microbiology, Santa Maria della Misericordia Hospital, Perugia, Italy

**Keywords:** fungal infections, antifungal resistance, pandrug, caspofungin, anidulafungin, epidemic, genomic surveillance, *Candida auris*

## Abstract

A difficult-to-control outbreak of *Candida auris* is ongoing in a large tertiary care hospital in Liguria, Italy, where it first emerged in 2019. In a retrospective analysis, 503 cases of *C. auris* carriage or infection were observed between July 2019 and December 2022. Genomic surveillance identified putative cases that no longer occurred as part of one defined outbreak and the emergence of echinocandin (pandrug) resistance following independent selection of *FKS1*^S639F^ and *FKS1*^F635Y^ mutants upon prolonged exposure to caspofungin and/or anidulafungin.

*Candida auris* is an emerging multidrug-resistant (MDR) pathogenic yeast associated with outbreaks of invasive infections in healthcare settings worldwide, recently classified by the World Health Organization (WHO) as a pathogen of 'critical priority’ [1]. Over the past decade, *C. auris* emerged independently across the globe and has been reported in more than 45 countries on six continents. Consistent with this global trend, the European Centre for Disease Prevention and Control (ECDC) recently reported a marked increase of infection or carriage of *C. auris* in Europe during the period 2019 to 2021, with several countries experiencing sporadic detections or hospital outbreaks, such as Italy [2,3].

Here we report on the evolution of a difficult-to-control outbreak of *C. auris* ongoing since 2020 in a large tertiary care hospital in Italy, where genomic surveillance recognised the independent emergence of echinocandin-resistant genotypes and proved useful in identifying cases that no longer occurred as part of one defined outbreak.

## Outbreak evolution

We performed a retrospective single-centre study investigating cases of *C. auris* at our hospital, hereafter referred to as HSM, a 1,200-bed teaching hospital representing the largest tertiary care facility in the region of Liguria, northern Italy, from 1 July 2019 to 31 December 2022. A case was defined as detection of *C. auris* from non-sterile and sterile body sites. Carriage was defined as the detection of *C. auris* from at least one non-sterile site (urine, skin and/or respiratory tract specimens) in the absence of clinical signs or symptoms of infection. Clinical isolates (infection) were those cultured to diagnose a disease state. Candidaemia was defined as illness in any patient who had *C. auris* isolated from at least one blood culture.

Following the first detection of a patient with candidemia and colonisation by *C. auris* in July 2019 at HSM [4], a subsequent increase in case numbers was recognised during 2020 in healthcare facilities in Liguria and in the neighbouring region of Emilia-Romagna [5]. Overall, at least 277 cases were reported in Liguria up to November 2021, mostly from our hospital, where multiple intensive care units (ICU) were affected. To contain the *C. auris* dissemination, a bundle of infection control interventions was implemented at HSM, including: (i) screening for skin carriage (combined axilla and groin skin swab) at admission to ICU for early identification of possible community-acquired cases; (ii) repeated weekly screening for carriage at skin, respiratory (whenever mechanically ventilated) and urine level during the ICU stay until first detection of *C. auris*; (iii) screening for skin carriage upon when a *C. auris*-negative patient was discharged from the ICU and admitted to a different ward, with preventive contact precautions pending culture results; and (iv) implementation of strict contact precautions for colonised patients.

**Figure 1:**
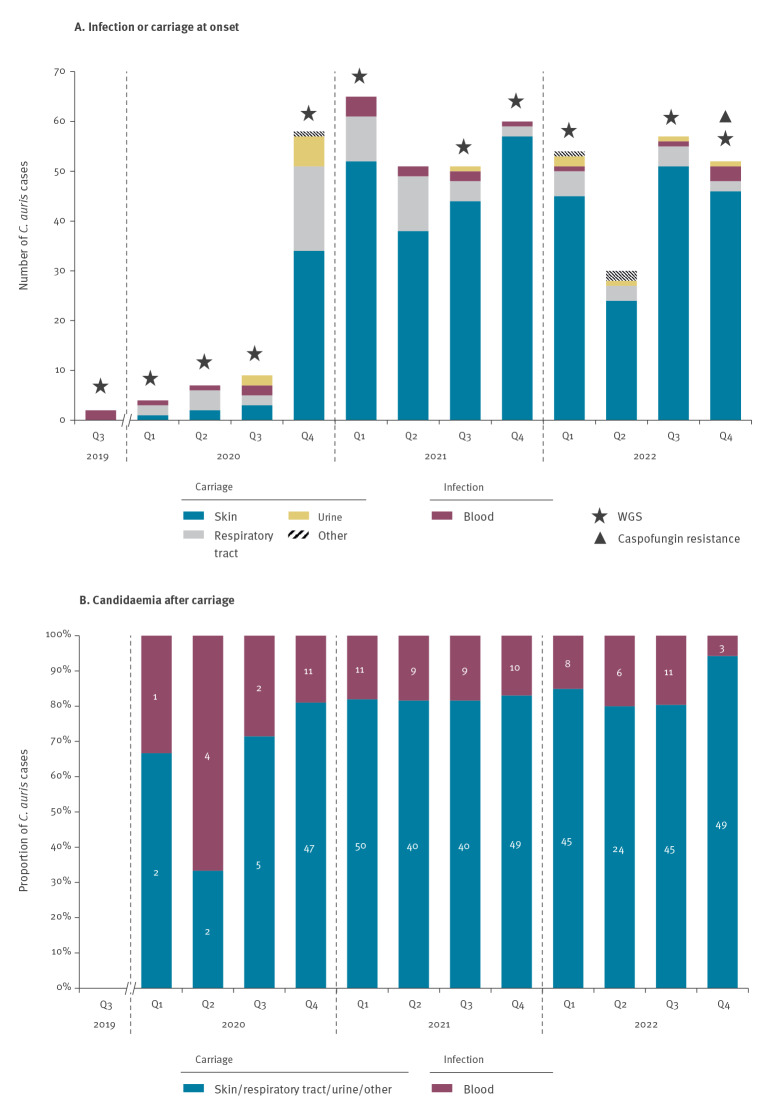
Retrospectively analysed cases of non-replicate *Candida auris* infection or carriage (n = 503) and proportions of carriage cases followed by candidaemia (n = 483), Italy, 2019–2022

The retrospective analysis of all first-occurring episodes of *C. auris* carriage or infection observed at HSM from the first detection of *C. auris* in July 2019 [5] to December 2022 yielded 503 cases (Figure 1A). Skin, respiratory or urine carriage was detected in 483 (96%) of the 503 cases, while first isolation from blood was registered in 20 (4%) cases (Figure 1A). Among the 483 colonised patients, 85 (17.6%) subsequently developed candidaemia (Figure 1B).

Q: calendar quarter; WGS: whole genome sequencing.

Panel B shows the case numbers on the columns.

Previous characterisation of *C. auris* isolates (n = 10) collected during the early phases of the outbreak (i.e. following the sharp rise in colonisation and infection cases registered during the first wave of COVID-19 pandemic) revealed that all belonged to the clade I (South Asian) and showed a uniform high-level resistance to fluconazole and amphotericin B [6]. However, identification of caspofungin-resistant infection isolates with pandrug-resistant (PDR) phenotypes, and of cases without epidemiological links to those ICU mainly involved in the epidemic, revealed the dynamic nature and continued evolution of the outbreak. As such, additional phenotypic (antifungal susceptibility testing (AFST)) and molecular (whole genome sequencing (WGS)) investigations were carried out on 32 *C. auris* isolates, including: (i) two isolates resistant to echinocandins, (ii) seven isolates from patients with confirmed *C. auris* carriage/infection from samples collected within 0–24 h since hospital admission (i.e. suspected epidemiologically unrelated cases) and (iii) 23 isolates representative of the outbreak timespan (Figure 1A). Details of the laboratory methods are appended in the Supplement. 

When available, isolates cultured from a different specimen from the same patient were also characterised (n = 18) to allow for pairwise genomic comparison between echinocandin-susceptible and -resistant isolates and between surveillance and clinical isolates (mean separating days: 38 ± 40; median: 23; interquartile range (IQR): 12–64). Overall, 50 *C. auris* isolates were subjected to further investigation, together with 10 *C. auris* isolates previously characterised during the emergence of the outbreak [6].

## Increased clonal heterogeneity among outbreak isolates

**Figure 2:**
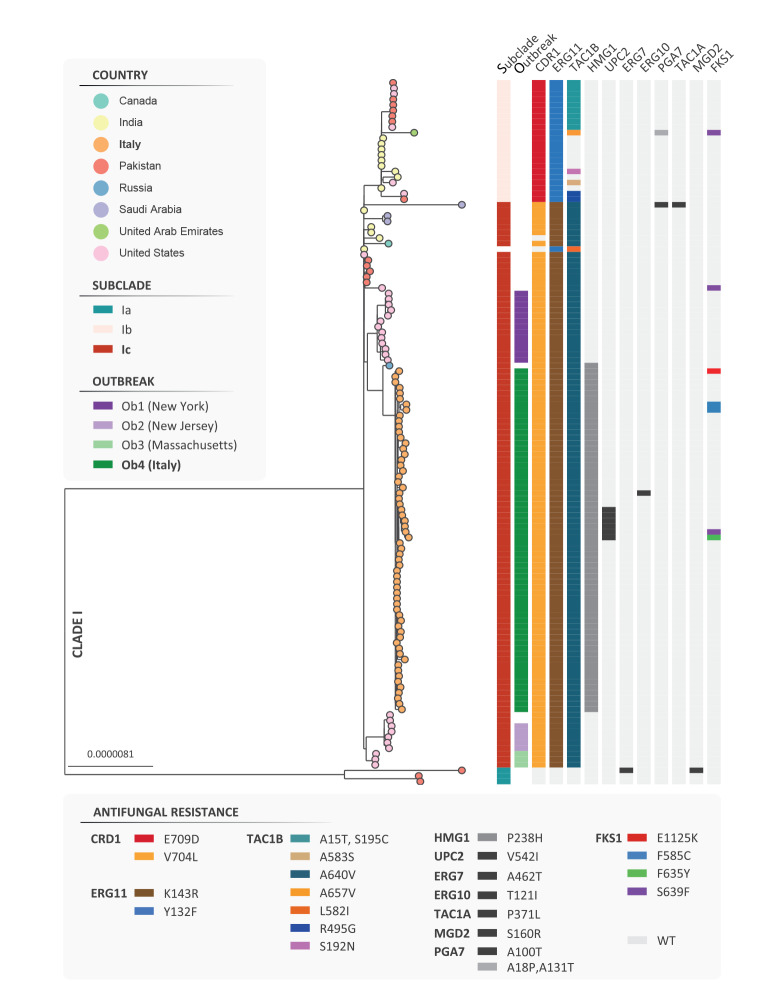
Maximum likelihood phylogeny of *Candida auris* outbreak isolates, Italy, 2019–2022 (n = 60) and from a benchmark dataset including cases (n = 64) from clade I (South Asian)

We performed a global core-genome single nucleotide polymorphism (SNP) phylogenetic analysis (see the Supplement for methodological details) including sequence data of *C. auris* isolates collected in the early (n = 10; 2019–2020) and late (n = 50; 2020–2022) stages of the outbreak recognised at HSM [6]. Among these 60 isolates from HSM, two were from patients initially diagnosed with *C. auris* infection or colonisation at two hospitals located in the same region (Liguria), who were later transferred to our facility. Sequence data of international isolates (n = 110) from validated outbreak benchmark datasets was also included in that analysis [7,8]. All outbreak isolates from HSM belonged to the clade I (South Asian), representing a monophyletic group (Ob4) within subclade Ic; outbreak cases from the United States (US) (Ob1–3) were reliably resolved within the same subclade (Figure 2). In Supplementary Figure S1, we provide results of a phylogenetic analysis carried out with a global collection of *C. auris* genomes for the clade assignation.

WT: wild type.

The colours of the isolate tips represent the country of isolation. Details about subclade designation [7,8], and genetic mutations associated with azoles (*CDR1, ERG11, TAC1B, HMG, UPC, ERG7, ERG10, PGA7, TAC1A, MGD2*) and echinocandins (*FKS1*) resistance are also provided.

Analysis of loci commonly involved in antifungal resistance revealed the presence of multiple alterations associated with the population structure at subclade level (e.g. *CDR1*^V704L^, *ERG11*^K143R^, *TAC1B*^A640V^). Furthermore, we identified a genetic signature uniquely represented in outbreak isolates from HSM (i.e. *HMG1*^P238H^). Screening for the *HMG1*^P238H^ allele using a comprehensive multinational dataset including 2,283 publicly available *C. auris* genomes revealed that this variant was detectable in a single record (i.e. excluding sequenced isolates from HSM), representing the first *C. auris* strain (RCPF-1821) isolated in Russia in 2017 [9]. Consistently, phylogenetic analysis resolved RCPF-1821 close to outbreak isolates from HSM (Figure 2). The Supplement lists the inclusion criteria adopted to establish the genome dataset and related accession numbers.

Evaluation of the global clonal heterogeneity among the 60 isolates from our facility revealed uneven separating SNPs (mean: 9 ± 5; median: 8; IQR: 5–12). Comparison of 18 carriage and clinical pairs of isolates from 18 patients yielded uniformly low values of separating SNPs (mean: 4 ± 4; median: 3; IQR: 2–6), defining a baseline genomic diversity among outbreak isolates. With respect to that baseline diversity, high values of separating SNPs were observed among five of seven isolates from cases suspected to be epidemiologically unrelated (mean: 14 ± 3; median: 13; IQR: 12–16). Further investigations revealed that four of the five patients had a recent history of contact with the healthcare system or transfer to our institution from other hospitals; no history of previous hospital admission was recorded for the fifth patient. Despite the initial classification, the remaining two of the seven isolates showed a SNP difference (mean: 6 ± 4; median: 6; IQR: 4–9) close to the estimated baseline genomic diversity, and retrospective inspection of clinical records revealed epidemiological links to the outbreak for those patients. Notably, further estimation of the overall clonal heterogeneity excluding the five cases suspected to be epidemiologically unrelated (i.e. among 55 outbreak isolates) yielded values of separating SNPs (mean: 5 ± 2, median: 5; IQR: 4–7) similar to those previously inferred as baseline genomic diversity (mean: 4 ± 4; median: 3; IQR: 2–6).

## Antifungal susceptibility profiles

We carried out AFST by broth microdilution following the Clinical and Laboratory Standards Institute guidelines and interpreted as per the US Centers for Disease Control’s tentative breakpoints (see the Supplement for the microdilution method and interpretation criteria) [10]. All tested isolates were resistant to amphotericin B and fluconazole and showed low MIC values for all other tested azoles but voriconazole (Table 1). The resistance profile for these antifungal agents was overall stable, consistent with susceptibility data obtained during the initial phase of the outbreak [6]. On the other hand, although most isolates retained susceptibility against echinocandins, evolution towards caspofungin resistance was observed in some cases, with a variable cross-resistance to micafungin and anidulafungin (Table 1).

**Table 1:** In vitro antifungal susceptibility profiles of *Candida auris* isolates characterised in this study, Italy, 2019−2022 (n = 60)

Antifungal agent	MIC range	MIC_50_	MIC_90_
Fluconazole	> 256	> 256	> 256
Itraconazole	0.25 to 2	0.5	1
Voriconazole	2 to 4	2	4
Posaconazole	0.25 to 1	0.25	0.5
Isavuconazole	1 to 2	1	2
Caspofungin	0.06 to > 8	0.25	0.25
Anidulafungin	0.125 to 4	0.25	0.5
Micafungin	0.125 to > 8	0.125	0.25
Amphotericin B	1 to 4	4	4

MIC: minimum inhibitory concentration.

MIC range, MIC_50,_ MIC_90_ values reported as mg/L.

## In vivo evolution to echinocandin resistance

In late 2022, two cases of caspofungin-resistant *C. auris* candidaemia were observed. Both patients had previously tested positive for skin carriage and experienced prolonged exposure to echinocandins due to intraabdominal candidiasis after abdominal surgery (Table 2). Resistant isolates emerged after 19 (Patient A) and 74 (Patient B) days of echinocandin exposure, both during anidulafungin administration that was subsequently replaced by liposomal amphotericin B and flucytosine (Patient A) and liposomal amphotericin B (Patient B). Both patients resolved *C. auris* candidaemia and were alive at the last follow-up. The sequential (collected within 23 and 74 days) echinocandin-susceptible (skin) and -resistant (blood) isolates were subjected to WGS to decipher the genetic bases of resistance (see the Supplement for details about sequence analyses) [6]. Comparative analyses revealed few (2–5) coding non-synonymous SNPs within each pair, and that both resistant isolates carried an altered *FKS1* allele with the F635Y (Patient A) or S639F (Patient B) substitution. Altogether, these findings suggested that echinocandin resistance resulted from antifungal pressure and emerged independently, following the selection of different *FKS1* genotypes (Table 2).

**Table 2:** In vitro antifungal susceptibility profiles of the sequential caspofungin-susceptible and ‑resistant *Candida auris* isolates associated with different *FKS1* genotypes, Italy, 2019−2022 (n = 2)

	Patient A		Patient B	
*FKS1* genotype	WT	F635Y	WT	S639F
Antifungal agent	MIC			
Caspofungin	0.06	> 8	0.25	2
Anidulafungin	0.125	2	0.5	4
Micafungin	0.125	2	0.125	> 8
Days to candidaemia from hospital admission	29		95	
Days to candidaemia from first *C. auris* colonisation	23		74	
Previous echinocandin exposure^a^	Cumulative days			
Anidulafungin	19		23	
Caspofungin	None		58	

MIC: minimum inhibitory concentration; WT: wild type.

^a^ Exposure to caspofungin preceded that with anidulafungin in patient B.

MIC range, MIC_50,_ MIC_90_ values reported as mg/L.

## Discussion

The ECDC reported in November 2022, that *C. auris* emerged progressively in Europe during 2020 and 2021, a consequence of sporadic (France, Germany) or multiple (Greece, Italy) outbreaks; regional endemicity was also reported (Spain) [2]. In most scenarios, however, available information uniquely relied on epidemiological data and lacked implementation of genomic surveillance to track the spread of *C. auris* and monitor evolution of the outbreaks. Here we have provided a genomic-informed snapshot of a large *C. auris* outbreak in Europe, gathering new insights about its clonal heterogeneity and in vivo evolution towards PDR phenotypes.

In Italy, the first *C. auris* outbreak was recognised in our facility during 2020 [4,6]. Despite the bundle of infection control interventions implemented at HSM to contain the *C. auris* dissemination, the epidemic proved difficult to control and additional cases were increasingly recognised throughout 2021 and 2022. Overall, 503 *C. auris* cases were identified from July 2019 to December 2022, mostly contributed by carriage, with a total of 105 cases of candidaemia. The ability to preferentially colonise cutaneous surfaces is a distinguishing feature of *C. auris*, leading to an increased risk of intrahospital transmission and development of candidaemia, which is considerably enhanced by the simultaneous colonisation of skin and other sites (multiple site colonisation) [11].

Genomic surveillance traced the HSM outbreak mainly to the clonal expansion of a single lineage, without evidence of phylogeographical mixing of multiple clades, and proved fundamental in dissecting the clonal heterogeneity of *C. auris*. Indeed, we identified distinctive genetic signatures (e.g. *CDR1*^V704L^, *ERG11*^K143R^, *TAC1B*^A640V^, *HMG1*^P238H^) that altogether could help to molecularly trace the dissemination of this lineage at national or international level.

Determination of SNPs separating paired carriage and clinical isolates from the same patient proved useful to infer the baseline genetic diversity in this outbreak, strengthening data obtained from the few similar evaluations carried out on sporadic cases in Australia and on outbreak cases in the US [12,13]. Using the estimate of the baseline genetic diversity as a point of reference to assess transmissions, we inferred that genetically heterogeneous subpopulations were present and that at least some cases no longer occurred as part of a defined outbreak in our setting. These findings suggest that *C. auris* could be more widely disseminated than expected, as the transfer of patients colonised by *C. auris* between facilities may have been unreported. The most recent evidence shows that *C. auris* has been detected in four Italian regions: Liguria, Emilia Romagna, Piedmont and Veneto [14]; nevertheless, regions other than Liguria notified only a limited number of cases (n = 64) in the period from November 2021 to December 2022. Concerns about possible establishment of *C. auris* as a healthcare-associated pathogen in Europe, as in the epidemiological scenario in the US [15], have recently been raised by the ECDC [2].

In addition to its ability to disseminate within the healthcare setting, *C. auris* received attention because of its MDR phenotype, frequently involving a reduced susceptibility to fluconazole (ca 80–90%) and amphotericin B (ca 23–30%), and sporadically to echinocandins (ca 3.5–1.2%) [3,16].

It is of concern that we identified echinocandin-resistant isolates which evolved in vivo upon prolonged exposure to anidulafungin or anidulafungin and caspofungin, which led to the independent emergence of different *FKS1* mutants. Unlike *FKS1*^S639Y^, commonly observed in echinocandin-resistant *C. auris* that have so far been reported [16,17], *FKS1*^F635Y^ represents a novel genotype recently identified in two isolates in India [18]. These alterations accounted for a distinct echinocandin-resistance profile and were shown to contribute a differential response to echinocandin treatment in a murine model of disseminated infection, suggesting that the *FKS1* genotype could be a more accurate predictor of treatment response than echinocandin MICs [18].

Our investigation has some limitations, since the absence of characterisation of isolates from healthcare facilities located in other regional areas prevented us from ruling out the introduction of multiple *C. auris* lineages, and from establishing representative genetic signatures of the clone(s) circulating in Italy.

## Conclusion

The evolution towards PDR phenotypes calls for close monitoring of antifungal resistance in patients with prolonged exposure to echinocandins. Prompt implementation of genomic surveillance and antifungal stewardship programmes is critical to contain the selection and spread of PDR *C. auris*.

## Supplementary Data

163000 Supplement
