# Supplementary material for: In vivo evolution to echinocandin resistance and increasing clonal heterogeneity in Candida auris during a difficult-to-control hospital outbreak, Italy, 2019 to 2022
Source: Euro Surveill. 2023 Apr 6;28(14):2300161. doi: 10.2807/1560-7917.ES.2023.28.14.2300161 (PMC10283462; doi:10.2807/1560-7917.ES.2023.28.14.2300161)
Supplement: Supplement [file 23-00161_DI-PILATO_Supplement.pdf]

This supplementary material is hosted by *Eurosurveillance* as supporting information alongside the article ***In vivo evolution of echinocandin resistance in Candida auris and increasing clonal heterogeneity during a difficult-to-control hospital outbreak, Italy, 2019 to 2022***, on behalf of the authors, who remain responsible for the accuracy and appropriateness of the content. The same standards for ethics, copyright, attributions and permissions as for the article apply. Supplements are not edited by *Eurosurveillance* and the journal is not responsible for the maintenance of any links or email addresses provided therein

Storge of fungal isolates and culturing conditions

All fungal isolates were stored at -80°C in Brain Heart Infusion broth (BHI) (Oxoid, Hampshire, UK) containing 20% v/v glycerol (Sigma Aldrich, Saint-Louis, Missouri, USA). The glycerol stocks were used as sources of the isolates for all the experiments performed in this study. From frozen stocks, isolates were plated on Sabouraud Dextrose plates (Oxoid, Hampshire, UK) and incubated overnight at 37 °C prior to downstream analyses (i.e., genomic DNA extraction, antifungal susceptibility testing).

Antifungal susceptibility testing (AFST)

Antifungal susceptibility testing of nine antifungal agents (i.e., amphotericin B, fluconazole, itraconazole, voriconazole, posaconazole, isavuconazole, caspofungin, anidulafungin, and micafungin) was performed by reference broth microdilution according to the CLSI M27-A3 guidelines [1], using commercial broth microdilution plates (Sensititre YeastOne, Thermo Scientific, Waltham, MA, USA) and the manufacturer's instructions. As *C. auris*-specific susceptibility breakpoints have not yet been established, tentative breakpoints proposed by the CDC (https://www.cdc.gov/fungal/candida-auris/c-auris-antifungal.html, accessed on March 8, 2023). In detail, resistance breakpoints were defined as follows: micafungin at ≥4 µg/mL, caspofungin at ≥2 µg/mL, anidulafungin at ≥4 µg/mL, amphotericin B at ≥2 µg/mL, fluconazole at ≥32 µg/mL.

Whole genome sequencing (WGS) and bioinformatics

Total genomic DNA was extracted from fungal cultures grown in YPD broth (Oxoid, Hampshire, UK), using the Qiagen DNeasy PowerLyzer PowerSoil Kit (Qiagen, Hilden, Germany). Shotgun libraries were prepared from purified genomic DNA samples and sequenced with the NovaSeq platforms (Illumina, Inc., San Diego, CA), using a 2x150 bp paired-end approach. Raw reads were assembled using SKESA (https://github.com/ncbi/SKESA). Global phylogenomics and pairwise chromosomal comparisons between caspofungin-susceptible and -resistant isolates was investigated by generation of core-genome single nucleotide polymorphisms (SNPs) using using BWA v0.7.17 to align the Illumina reads against the *C. auris* B8441 reference genome (Acc. no: PEKT00000000.2) [2], and NUCmer v3.1 and FreeBayes v1.1.0 to identify repetitive regions and call SNPs, respectively [3,4]. Maximum-likelihood phylogenetic trees were constructed on concatenated core-genome SNPs using IQ-TREE v1.6.12, using a general time reversible (GTR) nucleotide substitution model [5]. To perform comprehensive comparative analyses, a global *C. auris* genome dataset was established by retrieving raw reads data from the NCBI SRA database (https://www.ncbi.nlm.nih.gov/sra/?term=candida+auris, accessed on December 30, 2022) through the RunSelecter tool. A total of 2283 genome records (https://figshare.com/s/e5cbd1d278680073b7b6) were selected using the following inclusion criteria: assay type: WGS; organism: *Candida auirs*; host: homo sapiens; instrument: Illumina MiSeq, iSeq, HiSeq, NextSeq, NovaSeq; sequenced megabases: >200. The genome sequence of the *C. auris* strain B8441 (Acc. no: PEKT00000000.2) was used as reference to verify the presence of mutation possibly involved in antifungal resistance,

including: ERG1 (B9J08_000261), ERG10 (B9J08_003730), ERG11 (B9J08_001448), ERG12 (B9J08_005525), ERG13 (B9J08_004500), ERG2 (B9J08_004943), ERG20 (B9J08_003125), ERG24 (B9J08_003026), ERG25 (B9J08_000367), ERG26 (B9J08_000920), ERG27 (B9J08_004245), ERG3 (B9J08_003737), ERG4 (B9J08_002852), ERG5 (B9J08_002349), ERG6 (B9J08_005340), ERG7 (B9J08_005007), ERG8 (B9J08_001389), ERG9 (B9J08_004587), HMG1 (B9J08_003224), IDI1 (B9J08_001421), MVD (B9J08_001266), FKBP12 (B9J08_001031), CDR1 (B9J08_000164), TAC1B (B9J08_004820), TAC1A (B9J08_004819), UPC2 (B9J08_000270), MRR1 (B9J08_004061), FKS1 (B9J08_000964), PGA52 (B9J08_003903), PGA26 (B9J08_004577), CSA1 (B9J08_00195), HYR3 (B9J08_004100), PGA7 (B9J08_004469), MGD (B9J08_000656), MGD2 (B9J08_004828). Screening of mutated alleles within the global C. auris genome dataset was performed using KMA [6].

<u>Supplementary methods references</u>

[1] Clinical and Laboratory Standards Institute, editor. CLSI. Reference Method for Broth Dilution Antifungal Susceptibility Testing of Yeast. 4th Ed. CLSI standard M27. Wayne, PA: 2017.

[2] Li H, Durbin R. Fast and accurate long-read alignment with Burrows-Wheeler transform. Bioinformatics 2010;26:589–95. https://doi.org/10.1093/bioinformatics/btp698.

[3] Kurtz S, Phillippy A, Delcher AL, Smoot M, Shumway M, Antonescu C, et al. Versatile and open software for comparing large genomes. Genome Biol 2004;5:R12. https://doi.org/10.1186/gb-2004-5-2-r12.

[4] Garrison EG, Marth G. Haplotype-based variant detection from short-read sequencing. ArXiv Prepr 2012:q-bio.GN/1207.3907.

[5] Nguyen L-T, Schmidt HA, von Haeseler A, Minh BQ. IQ-TREE: a fast and effective stochastic algorithm for estimating maximum-likelihood phylogenies. Mol Biol Evol 2015;32:268–74. https://doi.org/10.1093/molbev/msu300.

[6] Clausen PTLC, Aarestrup FM, Lund O. Rapid and precise alignment of raw reads against redundant databases with KMA. BMC Bioinformatics 2018;19:307.

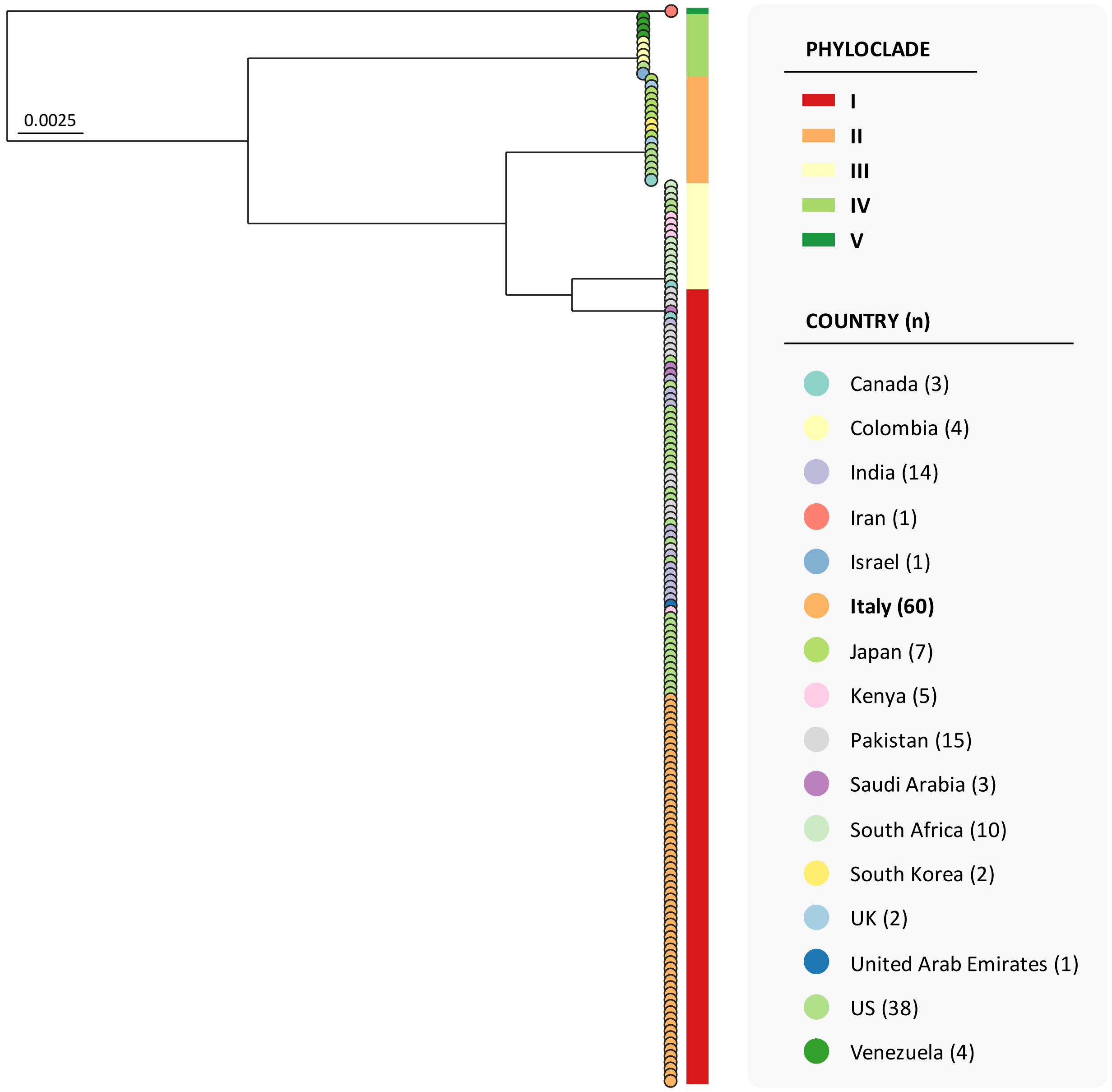

Supplementary Figure S1. Global phylogenetic analysis of *Candida auris* clustering in five major phyloclades. Maximum-likelihood phylogenetic tree of *C. auris* genomes characterized in this study (n=60) and representative genomes from different countries and clades (n=114), retrieved from previously established benchmarking datasets [7,8]. The colours of the isolate tips represent the different countries.
